# What Can Go Wrong When Applying Immune Modulation Therapies to Target Persistent Bacterial Infections

**DOI:** 10.33696/immunology.2.011

**Published:** 2020-01-03

**Authors:** Sofiya Micheva-Viteva, Elizabeth Hong-Geller

**Affiliations:** Bioscience Division, Los Alamos National Laboratory, Los Alamos, NM 87545, USA

**Keywords:** Chronic bacterial infections, Antibiotic tolerance, Immune modulation

## Abstract

Antibiotics can treat the acute phase of a disease, but often do not completely clear the etiologic agent, allowing the pathogen to establish persistent infection that can revive the disease in a frustrating recurrence of infection. The mechanisms that control chronic bacterial infections are complex and involve pathogen adaptations that favor survival from both host immune responses and antibiotic bactericidal activity. Often, the causative agents of persistent infections are not drug-resistant species. Instead, bacterial persister cells temporarily enter a physiological state that is refractory to different classes of antibiotics. Supplemental therapies that potentiate antibiotic bactericidal efficiency and/or immune clearance of persistent pathogenic species may greatly improve the outcome of infectious disease. Here, we discuss the various outcomes in experimental studies in which a mega-dose of the energy-boosting vitamin B3 (nicotinamide) was applied in murine models of chronic infection to stimulate immune clearance of chronic infection or as an immune prophylactic treatment against the highly infectious pathogen, *Burkholderia pseudomallei*. It is our intent to raise awareness of the risks associated with immune modulation therapies. There is great variance in host immune responses to pathogenic bacteria. Each immune modulation approach needs to be tailored to a well-characterized host-pathogen interaction.

## Introduction

Persistence is a transient phenotypic adaptation that confers survival to a small percentage of cells (between 0.01 and 10%) in genetically identical bacterial populations [1]. Nevertheless, persistence greatly affects the evolution of acquired drug resistance mutations by decreasing antibiotic efficacy and generating a reservoir of surviving cells that can contribute to the onset of chronic infectious disease [2–4]. Chronic illnesses range from the less malignant (e.g. nontuberculous pulmonary infection and strep throat) to the highly morbid with increased mortality rates upon relapse (e.g. latent tuberculosis and melioidosis) [5–8]. Usually, microbial drug resistance is the primary suspect. However, in many cases, relapse is caused by the same drug-sensitive pathogen that had initiated the acute phase of infection and had escaped both immune and therapeutic targeting through specific adaptation mechanisms [9–11].

### Antibiotic tolerance contributes to persistent bacterial infections

There are many complex mechanisms that govern the persistence phenotype. Bacteria survive the innate and primed acquired immunity by dampening their ability to activate immune responses. Such adaptations may include remodeling of bacterial surface antigens (e.g. lipopolysaccharide capsules), release of virulence factors that inhibit pro-inflammatory response and promote immune tolerance through dysregulation of the Th1-Th2 balance, evolution to an intracellular life cycle via phagosomal arrest, and inhibition of antigen presentation by MHC class II molecules of the infected host cells [7,8]. In the context of antibiotic tolerance, the mechanisms of persistence correlate with evolutionary adaptations by bacteria to resist toxic materials. Such adaptations include limited drug entry in microbial cells by generation of a protective coating composed of a complex assortment of lipids and glycolipids, activation of membrane transporters to enhance drug efflux, and stimulation of enzymes capable of detoxifying active oxygen and nitrogen radicals [1,12,13]. Although resilience to antibiotic bactericidal activity exists outside of the host environment (*in vitro*), attack of host innate immunity on bacterial invaders can further stimulate broad-spectrum drug tolerance [14–16]. Collectively, persister populations are a challenging target for antibacterial drugs and host immune defenses.

### Designing countermeasures to defeat bacterial persistence

Development of clinically-relevant therapeutics for eradication of chronic infections is a challenging task due to the multi-mechanistic nature of persistence. Complementation therapies with metabolites that enhance antibiotic bactericidal properties and concurrently boost the host immune response present a promising strategy to curtail chronic infections caused by bacterial persistence.

Bacterial persisters have a low level of metabolic activity [17–19]. Nicotinamide (NA), also known as Vitamin B3, is a precursor to NAD, an electron transfer metabolite (in the form of NADH) that plays a role in Respiratory Complex I, found in all living organisms from bacteria to humans. NA also serves as a precursor to NADPH, the catalytic unit of the NADPH oxidase enzyme complex, which generates superoxide free radical and reactive oxygen species (ROS) during a respiratory burst. In neutrophils and macrophages, NADPH oxidase function is crucial for killing invading bacteria [20]. Additionally, NA has the ability to reprogram the transcriptional profile of innate immune cells. Acting as a histone deacetylase inhibitor, NA can activate synthesis of antimicrobial peptides by neutrophils [21].

In a study published in the journal Infection and Immunity, we have evaluated the NA-assisted antibiotic killing of *Burkholderia* species in two experimental settings: (i) *in vitro* cultures of a clinical isolate of *B.thailandensis* CDC2721122 exposed to various metabolite and antibiotic concentrations in continuous flow pico-liter bioreactors and (ii) *in vivo* murine model of *B. pseudomallei* infection [22]. The model organisms featured high levels of persister populations *in vitro*. Furthermore, *B. pseudomallei* is a Select Agent pathogen that is notorious for establishing chronic melioidosis with a high percentage of relapse in the human host and a narrow window from symptom onset to death [6,23]. Although melioidosis is considered endemic to Southeast Asia, clinical cases have been reported globally, thus emphasizing the need to investigate advanced treatment options for emerging infectious diseases [24].

*In vitro* studies of bacterial persistence have many disadvantages when compared to investigation of chronic infections *in vivo*, due to oversimplification of the factors that stimulate persistence. Nevertheless, our *in vitro* platform based on microfluidic devices allowed us to subject bacterial cells to chemical time gradients and mechanical constraints that mimic blood circulation, conditions that are quite different from the standard laboratory techniques for bacterial growth under agitation or static conditions, which lead to progressive accumulation of secondary products of microbial metabolism. Additionally, the microfluidics devices enabled time-lapse observation of antibiotic and NA effects at a single cell level. Investigating the effect of each individual experimental parameter would have been technically or cost prohibitive in *in vivo* studies.

Our *in vitro* studies revealed that NA concentration is a key factor in the metabolite-assisted antibiotic activity against Burkholderia persister populations. NA potentiated the killing effect of various antibiotic classes at 4 μM, whereas we observed a bacteriostatic effect that expanded the persister population at concentrations above 20 μM. These observations were quite encouraging, in the context of a maximum daily dose of 35 mg that human adults can safely intake to gain NA plasma levels close to 6 μM [25]. Although NA-assisted antibiotic therapy significantly decreased *Burkholderia persisters in vitro*, it did not eliminate persisters completely. Therefore, we further investigated the effect of NA on antibacterial efficiency in the presence of human neutrophils, the first responders of host innate immunity. Pulse chase experiments showed that pre-treatment of human neutrophils with a high dose NA (50 μM) 24h prior to co-culture with bacteria in the microfluidic bioreactors containing antibiotic and 4 μM NA practically eliminated *Burkholderia* persister populations *in vitro*. These findings were in agreement with a model in which metabolite-assisted antibiotic therapy, combined with innate immune cells, could successfully eradicate persisters.

### Translating *in vitro* laboratory discoveries into animal studies

Eradication of *Burkholderia* persisters *in vitro* inspired further investigation of NA-assisted therapies for prevention of latent melioidosis in an *in vivo* mouse model of *B. pseudomallei* infection. Translation of experimental parameters from *in vitro* to *in vivo* studies may often yield unexpected results. Immunoprophylaxis with a high dose NA was the most efficacious regiment for eradication of *Burkholderia* persisters *in vitro*, but could this treatment be viable in a clinical setting? To achieve 50 μM NA in human plasma, one should receive 10 times the maximal daily dose, which is 350 mg daily. Most prescription medications with NA supplementation (e. g. high cholesterol and triglyceride medications) start at a total of 100 mg NA split between three doses taken throughout the day. According to the Mayo Clinic, a NA dose can be gradually increased to a maximum daily uptake of 1000 mg. Serious side effects may occur at 2000 g daily dose of NA. Theoretically, daily administration of 350 mg NA as an immunoprophylaxis against latent melioidosis seemed possible.

Eradication of chronic infections and treatment of multi-drug resistant bacterial infections through activation of innate or acquired immunity are therapeutic approaches proposed by many research groups. In fact, while designing the experimental regimen of effective NA concentration for prevention of chronic melioidosis in a mouse model, we found several studies that had reported successful application of NA as an antimicrobial agent against opportunistic pathogens, *Pseudomonas aeruginosa* and *Streptococcus pneumoniae*, and multi-drug resistant *Staphylococcus aureus* infections in mice [21,26]. NA was also found to ameliorate infection with *Citrobacter rodentium* in a mouse model of colitis (simulating *Helicobacter pylori* pathogenicity in primates) [27]. These studies demonstrated that NA selectively enhanced neutrophil killing of bacteria through direct stimulation of release of antimicrobial peptides. To achieve NA-augmented bacterial killing *in vivo*, a mega-dose of 250 mg/kg was applied in the mouse models [21,27], which roughly translated to 1 mM NA plasma concentration. A pilot study with NA in human patients with non-cystic fibrosis chronic inflammation of the lungs was also reported [26]. In an effort to achieve a clinically-effective NA concentration of 1mM, volunteers received a mega-dose of NA that exceeded not only the maximum daily dose, but also the safety limits of 2000 g daily. The maximum concentration of NA registered in the plasma of the human subjects in this study was 300 μM at 3000 g of NA daily. Activation of neutrophil markers was not detected in blood samples from subjects receiving this NA mega-dose, although enhanced killing of *S. pneumoniae* and *P. aeruginosa* was registered *ex vivo*. No information was shared regarding any side effects from the NA mega-dose treatment or any NA therapeutic effects for the patients with chronic lung inflammation.

### Stimulation of innate immune responses can be lethal to the host

In an attempt to prevent establishment of chronic melioidosis in mouse models of *B. pseudomallei* infection, we applied a mega-dose of NA, to serve both as an immunotherapeutic agent and a supplement to antimicrobial treatment with levofloxacin. The experimental group that received 250mg/kg of body weight NA as a sole therapy succumbed to infection at 100% compared to 50% death in the control group that received phosphate buffered saline with no antibiotic treatment. In contrast to studies where the same therapeutic dose of NA resolved infections with opportunistic pathogens [21,27], the NA-mediated stimulation of innate immune responses was lethal to animals infected with *B. pseudomallei*. Furthermore, the high dose of NA negatively affected antibiotic efficacy, increasing the persister population, as evidenced by extremely high microbial counts from the target organs (spleen, liver, and lungs) of animals that survived the acute phase of infection following antibiotic treatment and subsequent relapse of infection 10d after termination of therapy.

### To activate, or to suppress, that is the question

Depending upon the pathogen target, immunomodulation could be a viable option as an alternative therapeutic strategy or as a supplement to antimicrobial therapies. In opportunistic pathogens that have evolved immune suppressive adaptations, boosting innate immune responses was shown to reduce or eliminate infections. However, a similar approach has the potential to induce lethal immunopathology when applied to high-risk pathogens that stimulate an acute systemic inflammatory response (via induction of pyrexia, inflammation, and hyper production of ROS). In this latter scenario, it would be beneficial to use immune modulators that downregulate pro-inflammatory responses. Indeed, immune modulation with a COX-2 inhibitor that downregulated inflammation in *B. pseudomallei* infection was reported to significantly increase the survival of mice and to reduce bacterial load within the target organs [28].

When making a decision on the immunomodulation approach, to activate or suppress, we are highly dependent on the correct diagnosis and prior knowledge of the causative agent of disease. Unfortunately, the pathogen is not the only factor to consider in this complex equation. Understanding the host immune response for pathogen neutralization is an equally challenging factor for any immunomodulation therapy. An effective dose that is “just right” for the majority of test subjects could be lethal for a subpopulation of individuals with exaggerated innate immune responses that can cause systemic inflammation and organ failure. For example, pre-clinical tests are currently performed on animal models that are susceptible to infection by a particular pathogen. This susceptibility originates from genetic inheritance that drives specific innate and humoral immune responses. The BALB/c mouse strain is a predominant model used in pathogen infection studies due to increased susceptibility to microbial invasion. Compared to the C57BL/6 strain that normally survives higher lethal doses of pathogen challenge, the macrophages of BALB/c mice do not produce effector molecules for bacterial killing (e.g. lysosomal enzymes and nitric oxide). In an attempt to overcome this disadvantage, the innate immune system of BALB/c mice mounts exaggerated pro-inflammatory responses that result in systemic inflammation. To compensate for the enhanced systemic inflammation, the adaptive immune response in BALB/c mice assumes a Th2-type phenotype that supports establishment of chronic infections [29–31]. Similarly, genetic variation drives the diversity of human host responses to lethal infections.

Based on our observations, before safely applying any immunomodulation therapy on human subjects, a genetic test is needed to predict the individual’s response to therapy. The question is, do we have reliable genetic markers? Unless we can answer this question, immune modulation therapies for treatment of bacterial infections will remain a high-risk endeavor.
